# Feasibility of a multimodal ^18^F-FDG-directed lymph node surgical excisional biopsy approach for appropriate diagnostic tissue sampling in patients with suspected lymphoma

**DOI:** 10.1186/s12885-015-1381-z

**Published:** 2015-05-08

**Authors:** Stephen P Povoski, Nathan C Hall, Douglas A Murrey, Chadwick L Wright, Edward W Martin

**Affiliations:** 1 Division of Surgical Oncology, Department of Surgery, Arthur G. James Cancer Hospital and Richard J. Solove Research Institute and Comprehensive Cancer Center, The Ohio State University Wexner Medical Center, Columbus, OH 43210 USA; 2 Division of Molecular Imaging and Nuclear Medicine, Department of Radiology, The Ohio State University Wexner Medical Center, Columbus, OH 43210 USA; 3 Division of Nuclear Medicine and Clinical Molecular Imaging, Department of Radiology, Hospital of the University of Pennsylvania, Philadelphia, PA 19104 USA

**Keywords:** ^18^F-FDG, PET/CT, ^18^F-FDG-directed surgery, Real-time, Oncologic, Lymphoma

## Abstract

**Background:**

^18^F-FDG PET/CT imaging is widely utilized in the clinical evaluation of patients with suspected or documented lymphoma. The aim was to describe our cumulative experience with a multimodal ^18^F-FDG-directed lymph node surgical excisional biopsy approach in patients with suspected lymphoma.

**Methods:**

Thirteen patients (mean age 51 (±16;22–76) years), with suspected new or suspected recurrent lymphoma suggested by ^18^F-FDG-avid lesions seen on prior diagnostic whole-body PET/CT imaging, were injected IV with ^18^F-FDG prior to undergoing same-day diagnostic lymph node surgical excisional biopsy in the operating room. Various ^18^F-FDG detection strategies were used on the day of surgery, including, (1) same-day pre-resection patient PET/CT; (2) intraoperative gamma probe assessment; (3) clinical scanner specimen PET/CT imaging of whole surgically excised tissue specimens; (4) specimen gamma well counts; and/or (5) same-day post-resection patient PET/CT.

**Results:**

Same-day ^18^F-FDG injection dose was 14.8 (±2.4;12.5-20.6) millicuries or 548 (±89;463–762) megabecquerels. Sites of ^18^F-FDG-avid lesions were 4 inguinal, 3 cervical, 3 abdominal/retroperitoneal, 2 axillary, and 1 gluteal region subcutaneous tissue. Same-day pre-resection patient PET/CT was performed on 6 patients. Intraoperative gamma probe assessment was performed on 13 patients. Clinical scanner PET/CT imaging of whole surgically excised tissue specimens was performed in 10 cases. Specimen gamma well counts were performed in 6 cases. Same-day post-resection patient PET/CT imaging was performed on 8 patients. Time from ^18^F-FDG injection to same-day pre-resection patient PET/CT, intraoperative gamma probe assessment, and same-day post-resection patient PET/CT were 76 (±8;64–84), 240 (±63;168–304), and 487 (±104;331–599) minutes, respectively. Time from ^18^F-FDG injection to clinical scanner PET/CT of whole surgically excised tissue specimens was 363 (±60;272–446) minutes. Time from ^18^F-FDG injection to specimen gamma well counts was 591 (±96;420–689) minutes. Intraoperative gamma probe assessment successfully identified ^18^F-FDG-avid lesions in 12/13 patients. Histopathologic evaluation confirmed lymphoma in 12/13 patients and benign disease in 1/13 patients.

**Conclusions:**

A multimodal approach to ^18^F-FDG-directed lymph node surgical excisional biopsy for suspected lymphoma is technically feasible for guiding appropriate diagnostic tissue sampling of lymph nodes seen as ^18^F-FDG-avid lesions on diagnostic ^18^F-FDG PET/CT imaging.

## Background

Diagnostic ^18^F-fluorodeoxyglucose (^18^F-FDG) positron emission tomography/computed tomography (PET/CT) imaging is widely utilized in the clinical assessment of patients with lymphoma, including for initial staging, treatment monitoring during therapy, restaging after completion of therapy, and detection of suspected recurrent disease [1-13]. These various applications of diagnostic ^18^F-FDG PET/CT imaging can dramatically influence management/therapy recommendations for lymphoma patients and can have the potential to positively impact upon long-term patient outcomes.

The surgeon has continued to play an important role in facilitating the diagnostic pathway for patients with suspected new or suspected recurrent lymphoma, as the findings noted on diagnostic ^18^F-FDG PET/CT imaging that are considered suspicious for lymphoma frequently require the subsequent performance of a lymph node surgical excisional biopsy procedure for confirmation of a definitive diagnosis [14], as well as for histopathologic, immunophenotypic, flow cytometry, and molecular subtype analyses [15].

Over the last 15 years, there has been increasing interest by surgeons and nuclear medicine/molecular imaging physicians in utilizing ^18^F-FDG and PET/CT imaging technology to attempt to provide real-time information within the operative room and perioperative setting for cancer patients [16-75]. Specifically related to our collaborative efforts at The Ohio State University, we have previously investigated the use of a novel, multimodal imaging and detection approach involving perioperative patient and *ex vivo* surgical specimen ^18^F-FDG PET/CT imaging in combination with intraoperative ^18^F-FDG gamma probe detection [61]. In this regard, the aim of this study was to describe our cumulative experience in patients with suspected new or suspected recurrent lymphoma with a multimodal approach to ^18^F-FDG-directed lymph node surgical excisional biopsy for guiding appropriate diagnostic tissue sampling of lymph nodes that are seen as ^18^F-FDG-avid lesions on prior diagnostic whole-body ^18^F-FDG PET/CT imaging.

## Methods

All aspects of the current retrospective analysis were approved by the Cancer Institutional Review Board (IRB) at The Ohio State University Wexner Medical Center. The data for the current retrospective analysis were acquired from a master prospectively-maintained database (with database inclusion dates from June 2005 to June 2012), which were generated from the combination of several Cancer IRB-approved protocols, and which involved a multimodal imaging and detection approach to ^18^F-FDG-directed surgery for the localization and resection of ^18^F-FDG-avid lesions in patients with known and suspected malignancies. This multimodal imaging and detection approach to ^18^F-FDG-directed surgery included 166 patients who gave consent to participate in one of the IRB-approved protocols, and a total of 157 patients who eventually were taken to the operating room for ^18^F-FDG-directed surgery.

The imaging parameters of this multimodal imaging and detection approach to ^18^F-FDG-directed surgery have been previously described in significant detail elsewhere [61,74]. All participating patients received a same-day single-dose preoperative intravenous injection of ^18^F-FDG. All patients fasted for a minimum of 6 hours prior to receiving their same-day single-dose preoperative intravenous injection of ^18^F-FDG. Various ^18^F-FDG detection strategies were used, including: (1) same-day pre-resection whole body or limited field of view patient PET/CT scan (consisting of 6 to 8 field of view PET bed positions for whole body imaging with 2 minutes of PET imaging per each PET bed position or consisting of 1 to 3 field of view PET bed positions for limited field of view imaging with 2 minutes of PET imaging per each PET bed position); (2) intraoperative gamma probe assessment; (3) clinical scanner PET/CT imaging of whole surgically excised tissue specimens (consisting of 1 field of view PET bed position for 10 minutes); (4) specimen gamma well counting; and/or (5) same-day post-resection limited field of view patient PET/CT scan (consisting of 1 to 3 field of view PET bed positions, with 10 minutes of PET imaging per each PET bed position). The ^18^F-FDG PET/CT images were acquired on one of three clinical diagnostic scanners: (1) Siemens Biograph 16 (Siemens, Knoxville, Tennessee); (2) Phillips Gemini TF (Philips, Amsterdam, Netherlands); and (3) Siemens Biograph 64 Slice mCT (Siemens, Knoxville, Tennessee). The ^18^F-FDG PET/CT images were all analyzed on a Philips Extended Brilliance Work Station (Philips, Amsterdam, Netherlands). For each individual patient, the same-day preoperative diagnostic ^18^F-FDG PET/CT scan, the same-day postoperative diagnostic ^18^F-FDG PET/CT scan, and the whole surgically excised tissue specimen PET/CT scan were performed on the same clinical diagnostic scanner.

All presumed ^18^F-FDG-avid lesions seen on diagnostic ^18^F-FDG PET/CT imaging were reported out with a maximum standard uptake value (SUVmax) on each presumed ^18^F-FDG-avid lesion. Likewise, there was no “minimal set threshold value” for the SUVmax on any given presumed ^18^F-FDG-avid lesion to be considered as a designation of an “abnormal” hypermetabolic ^18^F-FDG-avid focus and to be reported out as “suspicious for malignancy”. Rather, the determination of any given presumed ^18^F-FDG-avid lesion to be considered as a designation of an “abnormal” hypermetabolic ^18^F-FDG-avid focus and to be reported out as “suspicious for malignancy” was considered more complex than simply being based upon its SUVmax, and also required the clinical judgment and expertise/experience of the reporting nuclear medicine physician.

Intraoperative gamma probe assessment was undertaken to all ^18^F-FDG-avid tissue sites using various combinations of 6 different available gamma detection probe systems that were synchronously or dissynchronously available during the study period. Intraoperative gamma probe assessment of ^18^F-FDG-avid tissue positivity was determined as based utilization of the concept of the three-sigma statistical threshold criteria. The three-sigma statistical threshold criteria for gamma probe positivity was previously popularized for radioimmunoguided surgery by Thurston [70,75-77], and the derivation of its application to ^18^F-FDG-avid tissue positivity was most recently articulated by Chapman et al. [75]. The three-sigma statistical threshold criteria was calculated by taking the standard deviation derived from the normal background tissue count rate and multiplying that standard deviation by a factor of three, and then adding that number to the normal background tissue count rate [70,75-77]. Using this statistical threshold methodology, intraoperative gamma probe assessment of ^18^F-FDG-avid tissue positivity was confirmed when the count rate for the target tissue exceeded the three-sigma statistical threshold criteria [70,75]. A fixed ratiometric ^18^F-FDG-avid lesion-to-background count ratio threshold was not utilized for the determination of probe positivity.

All continuous variables were expressed as mean value (± standard deviation; range). The software program IBM SPSS® 21 for Windows® (SPSS, Inc., Chicago, Illinois) was used for the data analysis. All mean value comparisons for continuous variables were performed by using the 2-tailed paired samples t-test. All categorical variable comparisons were made using 2 x 2 contingency tables that were analyzed by either the Pearson chi-square test or the Fisher exact test, when appropriate. P-values determined to be 0.05 or less were considered to be statistically significant.

## Results

There were 13 patients with either suspected new lymphoma (n = 2) or suspected recurrent lymphoma (n = 11) that were available for the current analysis, as suggested by ^18^F-FDG-avid lesions seen on their prior diagnostic whole-body PET/CT imaging. This included 10 females and 3 males. Their mean age was 51 (±16; range, 22–76) years. The mean SUVmax for the hottest ^18^F-FDG-avid lesion seen on their most recent prior diagnostic whole-body PET/CT scans was 11.5 (±8.0; range, 1.7-25.6). The time of the most recent prior diagnostic whole-body PET/CT scan to the time of surgery was 13 (±17; range, 0–48) days.

The anatomical sites of the ^18^F-FDG-avid lesions were 4 inguinal/groin region sites, 3 cervical region sites, 3 abdominal/retroperitoneal sites, 2 axillary region sites, and 1 gluteal subcutaneous tissue site. These anatomical sites of the ^18^F-FDG-avid lesions were clinically palpable on physical examination in only 5 of the 13 patients.

These 13 patients were intravenously injected with ^18^F-FDG prior to undergoing a same-day diagnostic lymph node surgical excisional biopsy procedure in the operating room, with a mean same-day ^18^F-FDG injection dose of 14.8 (±2.4; range, 12.5-20.6) millicuries or 548 (±89; range, 463–762) megabecquerels (Table 1).

**Table 1:** **Variables related to multimodal imaging and detection approach to** ^**18**^ **F-FDG-directed surgery**

Variable	Mean value (± SD; range)
Same-day ^18^F-FDG injection dose	14.8 (±2.4; 12.5-20.6) millicuries; or
	548 (±89; 463–762) megabecquerels
Time from ^18^F-FDG injection to same-day pre-resection patient PET/CT	76 (±8; 64–84) minutes
Time from ^18^F-FDG injection to intraoperative gamma probe assessment	240 (±63; 168–304) minutes
Time from ^18^F-FDG injection to same-day post-resection limited field of view patient PET/CT	487 (±104; 331–599) minutes
Time from ^18^F-FDG injection to clinical scanner specimen PET/CT of whole surgically excised tissue specimens	363 (±60; 272–446) minutes
Time from ^18^F-FDG injection to specimen gamma well counting	591 (±96; 420–689) minutes

All variables are expressed as mean value (± standard deviation; range).

Abbreviations: ^*18*^*F-FDG*^18^F-fluorodeoxyglucose, *PET/CT* positron emission tomography/computed tomography, *SD* standard deviation.

A same-day pre-resection patient PET/CT imaging was performed on 6 patients, including 4 same-day pre-resection whole body patient PET/CT scans and 2 same-day pre-resection limited field of view patient PET/CT scans. Intraoperative gamma probe assessment was performed on all 13 patients. Clinical scanner specimen PET/CT imaging of whole surgically excised tissue specimens was performed in 10 cases. Specimen gamma well counting was performed in 6 cases. A same-day post-resection limited field of view patient PET/CT imaging was performed on 8 patients.

As shown in Table 1, the time from ^18^F-FDG injection to same-day pre-resection patient PET/CT was 76 (±8; range, 64–84) minutes. The time from ^18^F-FDG injection to intraoperative gamma probe assessment was 240 (±63; range, 168–304) minutes. The time from ^18^F-FDG injection to same-day post-resection limited field of view patient PET/CT was 487 (±104; range, 331–599) minutes. The time from ^18^F-FDG injection to clinical scanner specimen PET/CT of whole surgically excised tissue specimens was 363 (±60; range, 272–446) minutes. The time from ^18^F-FDG injection to specimen gamma well counting was 591 (±96; range, 420–689) minutes.

Intraoperative gamma probe assessment successfully identified the ^18^F-FDG-avid lesion as probe positive (i.e., the ^18^F-FDG-avid lesion/target tissue count rate exceeded the three-sigma statistical threshold criteria) in 12/13 patients that were probed. Clinical scanner specimen PET/CT imaging of whole surgically excised tissue specimens verified ^18^F-FDG avidity within the sampled tissue from all 10 cases that were scanned. Same-day post-resection limited field of view patient PET/CT imaging verified successful removal of the intended ^18^F-FDG-avid lesions in all 8 patients that were scanned.

Histopathologic evaluation confirmed lymphoma in 12/13 patients and benign disease (i.e., florid follicular hyperplasia) in 1/13 patients.

**Figure 1:**
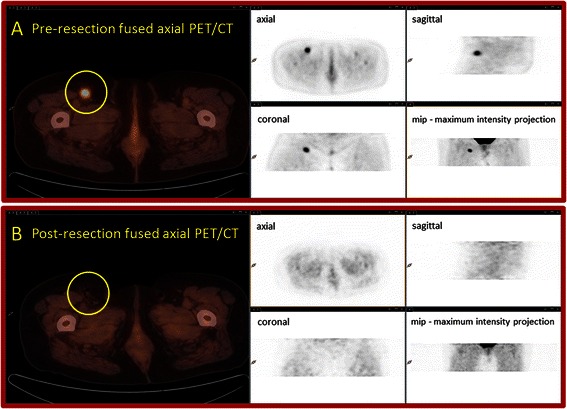
Patient imaging. **Panel A**: Same-day pre-resection whole body patient PET/CT scan images (i.e., fused PET/CT images and PET images) showing an isolated ^18^F-FDG-avid lymph node (seen within the region of the yellow circle on the fused axial PET/CT image) in the right inguinal region which was not palpable on clinical examination; and **Panel B**: Same-day post-resection limited field of view patient PET/CT scan images (i.e., fused PET/CT images and PET images) showing successful removal of the intended ^18^F-FDG-avid lymph node in the right inguinal region with residual air in the excision bed (seen within the region of the yellow circle on the fused axial PET/CT image).

**Figure 2:**
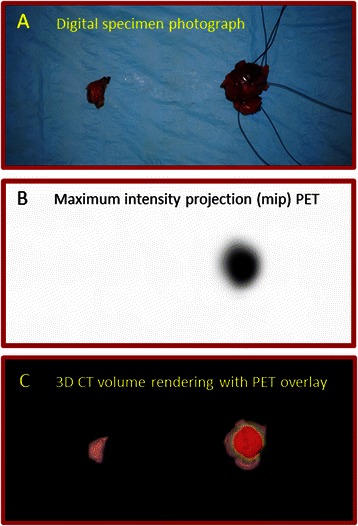
*Ex vivo* specimen imaging. **Panel A**: Digital specimen photograph of the *ex vivo* tissue specimens, for which the specimen on the right-hand side histologically was confirmed to contain angioimmunoblastic T-cell lymphoma, and for which the specimen on the left side represented normal adjacent fatty tissue; and **Panel B**: Maximum intensity projection (MIP) PET image of whole surgically excised tissue specimens, demonstrating ^18^F-FDG avidity within the specimen on the right-hand side, corresponding to the histopathology-proven lymphoma, and demonstrating no ^18^F-FDG avidity within the specimen on the left-hand side.; and **Panel C**: 3D CT volume rendering with PET overlay image of whole surgically excised tissue specimens, demonstrating ^18^F-FDG avidity within the specimen on the right-hand side, corresponding to the histopathology-proven lymphoma.

A representative example of a same-day pre-resection patient PET/CT scan and of a same-day post-resection patient PET/CT scan are shown in Figure 1 and of specimen PET/CT imaging of whole surgically excised tissue specimens is shown in Figure 2 for a specific case of histopathology-proven angioimmunoblastic T-cell lymphoma that originally presented as a non-palpable, ^18^F-FDG avid lesion within the right inguinal/groin region that was seen on a prior diagnostic patient PET/CT scan in a patient with a remote history of lymphoma.

## Discussion

For lymphoma patients, it is well recognized that ^18^F-FDG PET/CT imaging, in contrast to conventional anatomic imaging modalities (i.e., computed tomography, ultrasonography, or magnetic resonance imaging), allows for improved initial staging, treatment monitoring during therapy, restaging after completion of therapy, and detection of recurrent disease in the absence of any notably clinical and/or biochemical disease manifestations [1-13]. Along similar lines, ^18^F-FDG PET/CT imaging allows for the recognition of a lesser degree of disease burden than is detectable on conventional anatomic imaging modalities, thus creating a situation in which patients with suspected new or suspected recurrent lymphoma will less frequently present with palpable adenopathy on clinical examination. Surgical biopsy continues to represent the principal diagnostic pathway by which a definitive tissue diagnosis is confirmed in patients with suspected new or suspected recurrent lymphoma [14]. With the improved detection of more limited disease burden by ^18^F-FDG PET/CT imaging, which is more frequently associated with an inability to appreciate palpable adenopathy on clinical examination, the surgeon’s ability to successfully target the appropriate anatomical site of diagnostic tissue sampling for confirmation of the correct tissue diagnosis is made more challenging. Therefore, utilizing such innovative methodologies as a multimodal ^18^F-FDG-directed lymph node surgical excisional biopsy approach for guiding appropriate diagnostic tissue sampling of lymph nodes detected as ^18^F-FDG-avid lesions on diagnostic whole-body ^18^F-FDG PET/CT imaging can help the surgeon to maximize the likelihood of success in establishing the correct tissue diagnosis. Likewise, such methodologies have the potential for decreasing the degree of invasiveness as well as the degree of tissue disruption and tissue removal necessary for accomplishing successful tissue targeting.

Although we fully recognize that the current retrospective data analysis is based upon only 13 patients, the cases presented herein demonstrate that a multimodal ^18^F-FDG-directed lymph node surgical excisional biopsy approach for suspected lymphoma is technically feasible for guiding appropriate diagnostic tissue sampling of lymph nodes seen as ^18^F-FDG-avid lesions on diagnostic ^18^F-FDG PET/CT imaging, especially when such ^18^F-FDG-avid lesions are not easily appreciable as palpable adenopathy on clinical examination. Likewise, the conceptualization of this approach is well illustrated by the case presented in Figures 1 and 2. It is our belief that such a strategy can be best maximized when a multimodal imaging and detection approach is utilized involving perioperative patient and *ex vivo* surgical specimen ^18^F-FDG PET/CT imaging in combination with intraoperative ^18^F-FDG gamma probe detection. This line of reasoning has been validated by our group for a variety of disease-type-specific solid malignancies [16-18,33,35,36,38-41,43,44,46,47,52,61,69].

Along similar lines, three other groups of investigators have also previously described the successful utilization of commercially-available intraoperative hand-held gamma probe systems for ^18^F-FDG-directed lymph node surgical excisional biopsy in patients with suspected or documented lymphoma [27,31,50,67]. Gulec et al. described this technique in four patients in one report from 2006 [27] and in six patients in another report from 2007 [31]. Molina et al. described this technique in three patients in their report from 2009 [50]. Vos et al. described this technique in one patient in their report from 2012 [67]. However, none of these three other groups of investigators utilized perioperative patient and *ex vivo* surgical specimen ^18^F-FDG PET/CT imaging at the time of intraoperative ^18^F-FDG gamma probe detection, thus falling short of incorporating a multimodal approach to this sometimes challenging diagnostic dilemma.

The ability to successfully perform an ^18^F-FDG-directed lymph node surgical excisional biopsy procedure for suspected lymphoma is highly dependent upon the commercially-available intraoperative hand-held gamma probe that is used during such a surgical case. The most important performance parameters related to any given gamma detection probe system are generally thought to be: (1) overall sensitivity (i.e., efficiency); (2) spatial selectivity (i.e., radial sensitivity distribution); (3) spatial resolution (i.e., lateral sensitivity distribution); (4) energy resolution (i.e., spectral discrimination); and (5) contrast [46]. The most widely utilized commercially-available intraoperative hand-held gamma probes are generally designed for detecting radioisotopes of gamma-ray energies in the low-energy emission (0 keV to 150 keV) range and medium-energy emission (150 keV to 400 keV) range, thus allowing successful detection of radioisotopes such as: (1) technetium-99 m (^99m^Tc; 140 and 142 KeV; most commonly used for sentinel lymph node biopsy procedures and parathyroid surgery); (2) indium-111 (^111^In; 171 and 247 KeV; used with octreotide to detect neuroendocrine tumors); (3) iodine-123 (^123^I; 159 KeV; used with metaiodobenzylguanidine to detect neuroblastomas and pheochromocytomas); and (4) iodine-125 (^125^I; 35 KeV; previously used with anti-TAG-72 monoclonal antibodies and anti-CEA monoclonal antibodies during radioimmunoguided surgery) [46,70].

However, most commercially-available intraoperative hand-held gamma probes are not specifically designed to directly or indirectly detect the resultant 511 KeV gamma emissions following positron annihilation emanating from higher energy gamma photon emitting/positron emitting radionuclides such as fluorine-18 (^18^F) or iodine-124 (^124^I) [46,70]. Resultantly, there has been a recent appearance of commercially-available intraoperative hand-held gamma probes that are specifically intended for attempting to detect 511 KeV gamma emissions from higher energy gamma photon emitting/positron emitting radionuclides. These commercially-available intraoperative hand-held gamma probes have generally been designated as “PET” probes. The overall weight and physical size of any typical “PET” probe is generally a function of the degree of physical side shielding/collimation necessary to theoretically block adjacent background radiation, to limit the field of view, and to collimate the head of the probe, with the intention of limiting the area of tissue contributing to the probe count rate and of providing better spatial resolution between areas of tissue of differing radioactivity levels [46,70,78]. Attempts at improving the current “PET” probe design by further increasing physical side shielding/collimation or by increasing crystal diameter/thickness to capture a higher percentage of 511 KeV gamma emissions would only result in a “PET” probe configuration prohibitively large in physical size, heavy in weight, and which would be much more costly [46,70,78], thus representing significant obstacles to applying the currently commercially-available “PET” probes to the detection of ^18^F-FDG-avid tissues.

In order to attempt to bypass these physical barriers related to the degree of physical side shielding/collimation or crystal diameter/thickness in designing intraoperative hand-held gamma probes that are specifically intended for attempting to detect 511 KeV gamma emissions from higher energy gamma photon emitting/positron emitting radionuclides, engineering efforts have moved toward other directions for the adaptation of more novel “PET” probe designs that do not rely upon increasing physical side shielding/collimation or increasing crystal diameter/thickness. Examples of such alternative design concepts that can be adapted to “PET” probe design are secondary K-alpha x-ray fluorescence [70,78], active electronic collimation [28,54,58,60,67,79-81], and other crystal geometry designs using multiple small crystals with specific novel geometric configurations [82,83] for optimizing background rejection capabilities. These innovative alternative design schemas for detecting higher energy gamma photon emitting/positron emitting radionuclides, some of which have already been successfully applied to intraoperative hand-held gamma probe designs, are also the focus of current pre-clinical research that is actively looking at developing small platform, portable perioperative and intraoperative patient and *ex vivo* surgical specimen imaging devices with similar capabilities for detecting higher energy gamma photon emitting/positron emitting radionuclides. However, such small platform, portable perioperative and intraoperative patient and *ex vivo* surgical specimen imaging devices have not yet been fully realized or made commercially available for use in the clinical arena.

## Conclusions

A multimodal approach to ^18^F-FDG-directed lymph node surgical excisional biopsy for suspected lymphoma is technically feasible for guiding appropriate diagnostic tissue sampling of lymph nodes seen as ^18^F-FDG-avid lesions on prior diagnostic whole-body ^18^F-FDG PET/CT imaging. This multimodal approach can be very helpful to the surgeon for accomplishing successful targeting of the appropriate anatomical sites (corresponding to ^18^F-FDG-avid lesions) for successful diagnostic tissue sampling in confirming the correct tissue diagnosis in challenging cases involving patients with non-palpable suspected new or suspected recurrent lymphoma.
